# Comparative Proteomic Analysis of *Pleurotus ostreatus* Reveals Great Metabolic Differences in the Cap and Stipe Development and the Potential Role of Ca^2+^ in the Primordium Differentiation

**DOI:** 10.3390/ijms20246317

**Published:** 2019-12-14

**Authors:** Weiwei Zhu, Jinbo Hu, Yang Li, Bing Yang, Yanli Guan, Chong Xu, Fei Chen, Jingliang Chi, Yongming Bao

**Affiliations:** 1 School of Bioengineering, Dalian University of Technology, Dalian 116024, China; zhuweiwei@vip.163.com; 2 Microbial Research Institute of Liaoning Province, Chaoyang 122000, China; liyang_0223@163.com (Y.L.); ylguan2008@163.com (Y.G.); sss646@163.com (C.X.); chenfei3033@vip.sina.com (F.C.); chijingliang@sina.com (J.C.); 3 Laboratory of Photosynthesis and Environment, CAS Center for Excellence in Molecular Plant Sciences, Shanghai Institute of Plant Physiology and Ecology, Chinese Academy of Sciences, Shanghai 200032, China; hujinbo@sibs.ac.cn (J.H.); Yangbing@sibs.ac.cn (B.Y.); 4 School of Food and Environmental Science and Technology, Dalian University of Technology, Panjin 12421, China

**Keywords:** *Pleurotus ostreatus*, primordium differentiation, sphingolipid, Ca^2+^, metabolism, fruiting body, stipe

## Abstract

*Pleurotus ostreatus* is a widely cultivated edible fungus around the world. At present, studies on the developmental process of the fruiting body are limited. In our study, we compared the differentially expressed proteins (DEPs) in the stipe and cap of the fruiting body by high-throughput proteomics. GO and pathway analysis revealed the great differences in the metabolic levels, including sucrose and starch metabolism, and sphingolipid signaling and metabolism, and the differences of 16 important DEPs were validated further by qPCR analysis in expression level. In order to control the cap and stipe development, several chemical inducers were applied to the primordium of the fruiting body according to the pathway enrichment results. We found that CaCl_2_ can affect the primordium differentiation through inhibiting the stipe development. EGTA (ethyleneglycol bis (β-aminoethyl ether)-N,N,N′,N′-tetraacetic acid) treatment confirmed the inhibitory role of Ca^2+^ in the stipe development. Our study not only shows great metabolic differences during the cap and stipe development but also reveals the underlying mechanism directing the primordium differentiation in the early development of the fruiting body for the first time. Most importantly, we provide a reliable application strategy for the cultivation and improvement of the *Pleurotus ostreatus*, which can be an example and reference for a more edible fungus.

## 1. Introduction

*Pleurotus ostreatus* is the earliest edible fungus cultivar domesticated and cultivated by human beings [1]. Although the whole fruiting bodies of the *Pleurotus ostreatus* are edible, the cap has a better taste and is rich in nutrition [2]. Therefore, the fruiting bodies with short stipes are more popular in the market, and their price is higher. At present, research on the developmental mechanism of the *Pleurotus ostreatus* fruiting body is very limited. In the process of cultivation, the cap and stipe development of the fruiting body mainly depends on the environmental conditions [3]. For example, high ventilation and low carbon dioxide concentration can stimulate the primordium to differentiate toward the cap and inhibit the growth of stipe. However, during the production, high ventilation can reduce the humidity of the environment and reduce the growth rate, resulting in the cracked phenotype, which can severely influence the sales. Therefore, a reliable method for the *Pleurotus ostreatus* is urgently needed in the process of cultivation.

Fruiting body formation of the fungi is quite a complex process that starts with the hyphal knot formation in the dark and is followed by light-induced aggregation into compact secondary hyphal knots (fruiting body initials or primordium) from which the cap and stipe begin to differentiate according to their own destiny on the normal day–night cycle [4]. Therefore, primordium is more sensitive to environmental conditions, which can severely affect its further differentiation. For example, the further light signal is needed for the cellular differentiation of the primordium; otherwise, the primordium will develop into the elongated structure with an undeveloped cap [4,5,6]. This phenotype resembles the etiolated plant seedlings without light-mediated photomorphogenesis, and we also call it etiolated stipe. Stipe elongation is mainly a process of manifold cell elongation, rather than cell division, which is different from animals [4,7,8,9,10]. Although mature fruiting bodies of different species differ greatly in morphology, the genes regulating their development are highly conserved [11,12,13,14,15,16,17,18]. Previous studies have reported some genes involved in the fruiting body development in some species in Basidiomycete [11,19,20,21,22,23]. Research on *Pleurotus ostreatus* fruiting body development, especially on stipe elongation, is very limited.

With the rapid development of the sequencing technology, more macro-fungi genomes have been sequenced, including the *Pleurotus ostreatus* [14,24,25]. Sequencing information gives us a better annotation of the genome, especially of the protein-coding genes, which can provide a better tool to solve the agricultural problems from the molecular level [16,26,27,28,29]. Therefore, we attempted to investigate the developmental mechanism controlling the cap and stipe development by analyzing the differentially expressed proteins (DEPs). Through isobaric labeled quantitative proteomics, we got 373 DEPs from the cap and stipe. Bioinformatics analysis showed that the DEPs were mainly distributed in the membrane part and involved in many catalytic processes. Pathway enrichment analysis further revealed the key pathways in regulating the cap and stipe development, including the starch and sucrose metabolism, sphingolipid signaling, and metabolism pathways. In order to confirm the proteomic results, we further validate the expression of relative genes of the DEPs by qPCR analysis. Our results revealed that there are great metabolic differences during the cap and stipe development, indicating that the potential mechanism controlling the different developmental fates of the cap and stipe may be derived from the metabolic process.

In order to find a method to control the cap and stipe development, we chose four chemical inducers according to the pathway enrichment results. Finally, we found that CaCl_2_ application on the primordium of the fruiting body can effectively inhibit the development of the stipe. To clarify the mechanism, we treated the primordia with calcium ion chelator EGTA. Compared with the control, EGTA treatment produced a longer stipe phenotype, indicating a key role of endogenous Ca^2+^ on the primordium differentiation and stipe development. Notably, we proposed the potential mechanism through which the exogenous Ca^2+^ inhibits the stipe development, and the indispensable role of the sphingolipids in Ca^2+^ mediated growth and development.

## 2. Results

### 2.1. Isobaric Labeled Quantitative Proteomics Enabled the High-Throughput Proteomic Analysis of the Pleurotus ostreatus Fruiting Body

**Figure 1:**
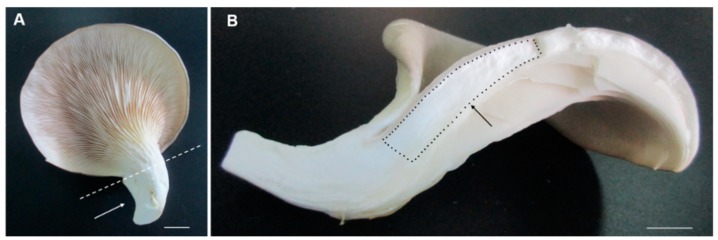
The cap and stipe of the fruiting body used in the proteomic study. White arrow (**A**) points to the stipe and the black dots region; (**B**) shows the cap without hymenium we used in the experiment. Bars = 1 cm.

**Figure 2:**
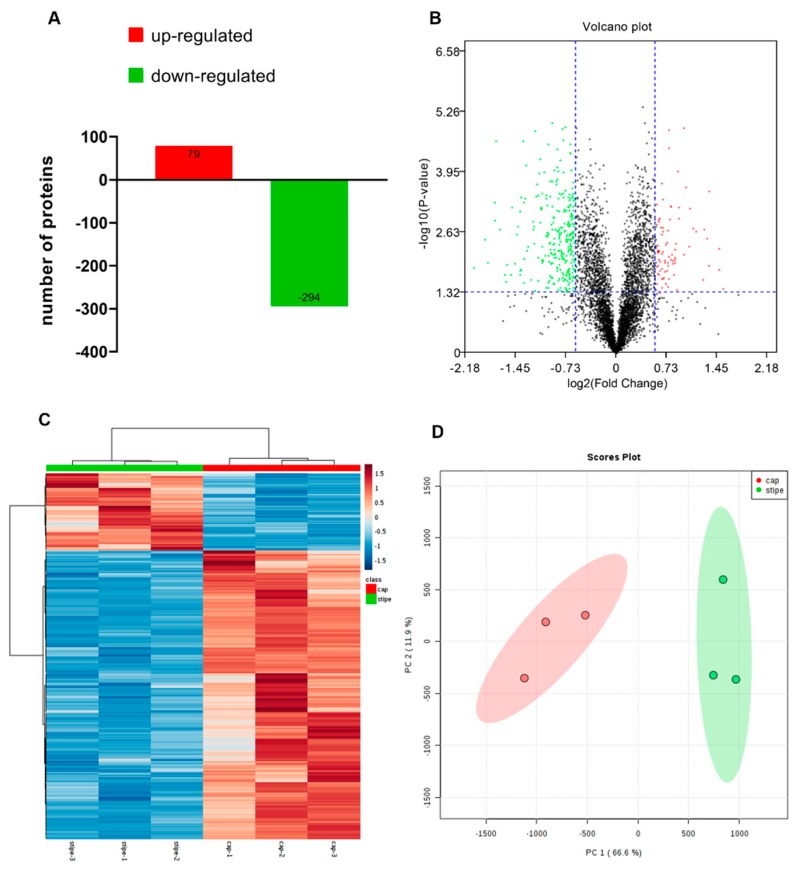
Statistical overview of the proteomic results. The total number of DEPs between the cap and stipe (**A**), and the volcano plot (**B**) shows the upregulated proteins (red dots) and downregulated proteins (green dots). The heatmap (**C**) shows the relative expression of the DEPs of each biological replicate. PCA plot (**D**) shows the clusters of six samples based on their similarity.

In order to investigate the different developmental mechanisms of the cap and the stipe, we used the iTRAQ technique to analyze the differentially expressed proteins in the two tissues (Figure 1A,B). A total of 4659 proteins from 106,679 PSMs and 30,524 peptides were identified (FDR of protein and PSM < 0.05), and, finally, 373 DEPs were obtained with fold change more than 1.5 or less than 0.67, and *p*-value < 0.05 (Figure 2A–C). Compared with the cap, there are 79 upregulated and 294 downregulated proteins in the stipe (Tables S1 and S2). Principal component analysis (PCA) showed good repeatability of each biological replicate (Figure 2D). The detailed information, including the accession number, fold change, *p*-value, and coverage of all the identified proteins, is listed in Table S3.

### 2.2. GO Analysis Revealed Great Differences in Membrane Part and Catalytic Activity

**Figure 3:**
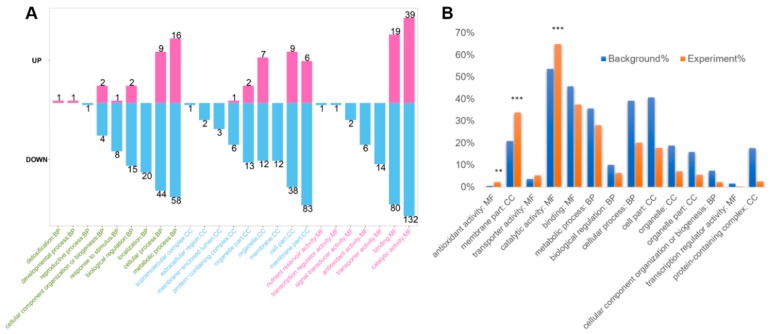
GO and GO enrichment analysis of the DEPs. (**A**) GO annotation with upregulation and downregulated proteins in level 2. (**B**) GO enrichment analysis of the DEPs (*p* < 0.05). Two asterisks indicate *p* < 0.01. Three asterisks indicate *p* < 0.001.

GO analysis can provide general information on the basic function of the proteins, especially for the non-model organisms in three aspects of biological process (BP), molecular function (MF), and cellular component (CC). Through GO annotation, the DEPs were categorized into different definition terms according to their functional domain. Each term was finally classified into a level 2 definition, summarized by the BP, MF, and CC. In our experiment, the BP was mainly distributed in the terms of metabolic process, cellular process, and the localization process. For the CC, the DEPs were mainly distributed in the membrane part, cell part, and organelles. The MF was mainly concentrated in the catalytic, binding, and transporter activity, according to the number of proteins in each term (Figure 3A). The GO annotation results indicated that there are great catalytic differences within the cells of the cap and stipe. The membrane system, especially the organelles with inner and/or outer membranes, may play a substantial role in maintaining the differentiation status of different cell types. Functional enrichment analysis (Figure 3B) further revealed a significant role of DEPs with membrane part and catalytic activity in the cap and stipe development (*p* < 0.001). The corresponding proteins and their annotations, expression ratio, and *p*-value in the two terms were listed in Table S4. This result is consistent with the GO annotation (Figure 3A), suggesting that the membrane system may play a key role in directing the cap and stipe differentiation.

### 2.3. KEGG and Protein–Protein Interaction Analysis Revealed the Significant Metabolic Differences Between the Cap and Stipe

**Figure 4:**
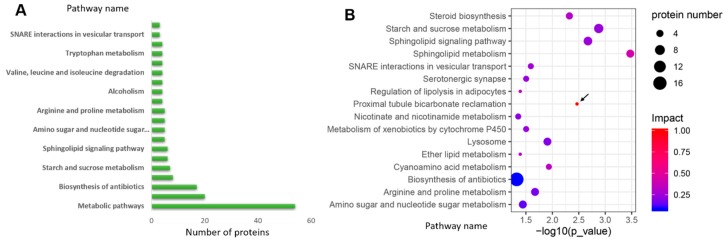
KEGG and KEGG enrichment analysis of the DEPs. (**A**) Top 20 of the DEPs involving pathways. (**B**) KEGG enrichment analysis of the DEPs (*p* < 0.05).

After primary mapping in the KEGG database, the pathways related to the metabolism rank highest among all (Figure 4A). Further enrichment analysis showed that 16 pathways (*p* < 0.05) were significantly enriched (Figure 4B). Among the enriched pathways, we found that 10 of them are involved in different biosynthesis and metabolic process. The most significant three pathways (*p* < 0.01) are involved in sphingolipid metabolism, starch and sucrose metabolism, and the sphingolipid signaling pathway (Figure 4B). The detailed protein information, including the annotation, ratio, and *p*-value, is listed in Table 1.

**Figure 5:**
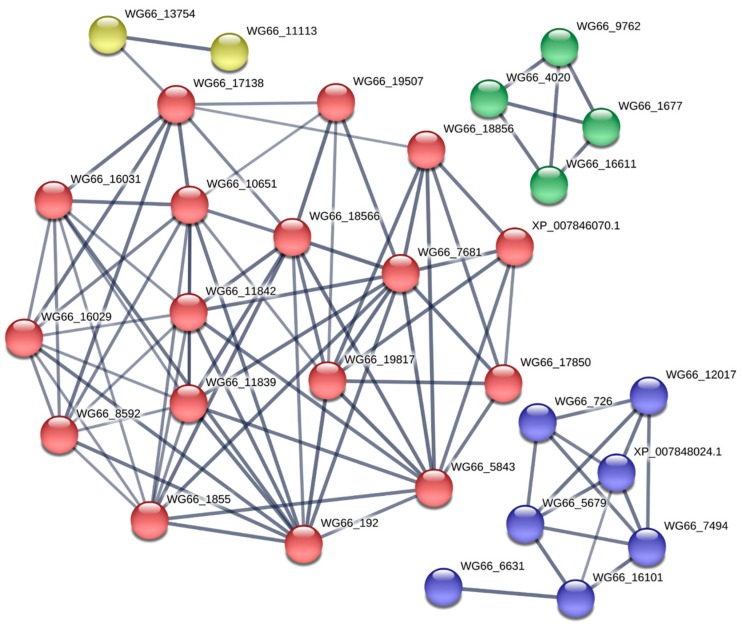
Protein–protein interaction analysis of the DEPs. Proteins in the significantly enriched pathways and their direct interactive partners. Red bubbles show the starch and sucrose metabolism pathway, blue bubbles represent the sphingolipid metabolism pathway, green bubbles represent the glycerophospholipid metabolism pathway, and the yellow bubbles represent the autophagy pathway.

To explore more information of the DEPs in the significantly enriched pathways, we analyzed their potential interactive partners across the fungi genomes in the STRING database. We found that all the DEPs and their direct interactors worked in an active collaboration with complex interactions (Figure 5). Although these interactions introduced more information, they were tightly concentrated in four pathways: starch and sucrose metabolism (red), sphingolipid metabolism (blue), glycerophospholipid metabolism (green), and autophagy (yellow). These results further indicate that there are great metabolic differences during the cap and stipe development. The potential interactive partners are shown in Table S5.

### 2.4. Quantitative Real-Time PCR Validation of the Expression of the DEPs

**Figure 6:**
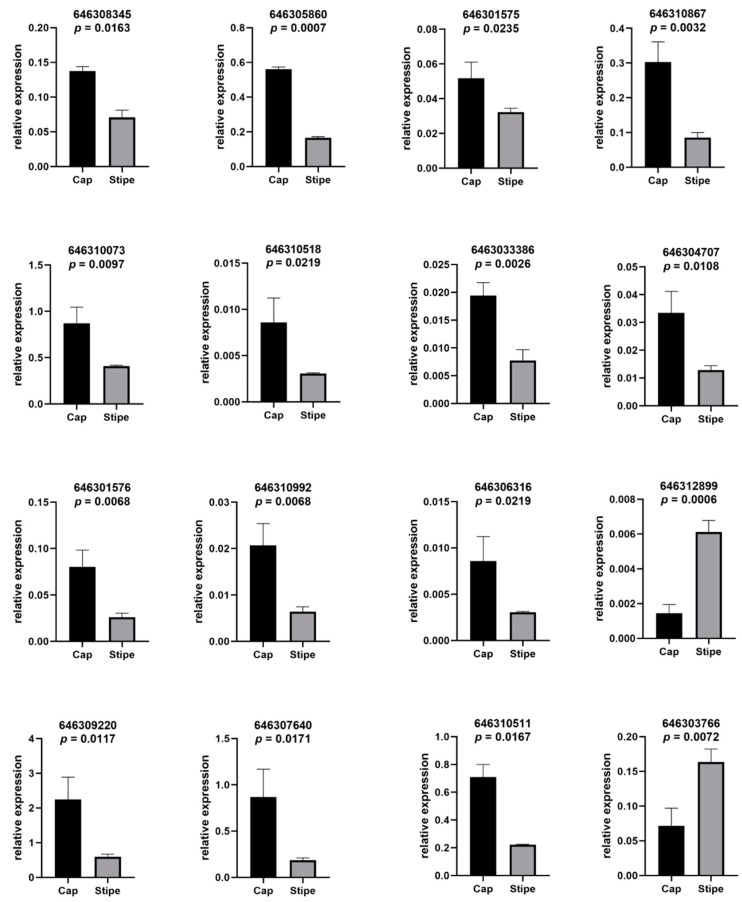
Quantitative real-time PCR validation of the DEPs in the proteomic results. Quantitative real-time PCR of the DEPs in the proteomic results. The accession number of the proteins and *p*-value (unpaired two-tailed *t*-test) is shown in each chart.

In order to verify the accuracy of our proteomic results, we used the quantitative real-time PCR to validate 16 of the differentially expressed genes (DEGs) in the iTRAQ results, including the sphingolipid metabolism and signaling pathways and other DEGs. The expression trend of 15 DEGs are in accordance with their proteomic results, except for the gene (646310867) in the sphingolipid-signaling pathway (Figure 6). This result suggested the reliability of our proteomic result, which will provide a precise foundation for future research and application. On the other hand, we noticed that the gene in the sphingolipid-signaling pathway showed opposite expression with the proteomic result, indicating that a complex and multilevel regulatory network of the signaling molecule existed during the signal transduction, such as the post-transcriptional or post-translational regulation.

### 2.5. Ca^2+^ Plays a Regulatory Role in the Primordium Differentiation

The whole fruiting body of *Pleurotus ostreatus* is mainly composed of the cap and the stipe, which are closely coordinated tissues differentiated from the primordium. We raised the question of whether this developmental process could be controlled by exogenous inducers, such as chemicals during the early developmental stage. We therefore focused on the most significantly enriched pathways and attempted to find some common characteristics of them, especially related to the chemical inducers. As a second messenger, more studies have shown that Ca^2+^ is involved in sphingolipid signaling and metabolism; thus, it plays a key role in many aspects of development [30,31,32,33]. The proximal tubule bicarbonate reclamation pathway has the highest enrichment rate (impact = 1.0) among all the pathways (Figure 4B, black arrow). Although it is a signaling pathway mainly in higher organisms, we believe that similar mechanisms may exist across the evolution of eukaryotes. There are also reports about the effects of plant hormones on fungi development [34,35,36,37]. Considering the above analysis, we chose 1 mM of calcium chloride (CaCl_2_), 1 mM of sodium bicarbonate (NaHCO_3_), 0.01 mM of Indole-3-acetic acid (IAA), and 0.01 mM of gibberellin 3 (GA3) as the exogenous inducer to apply on the primordium of the fruiting body.

**Figure 7:**
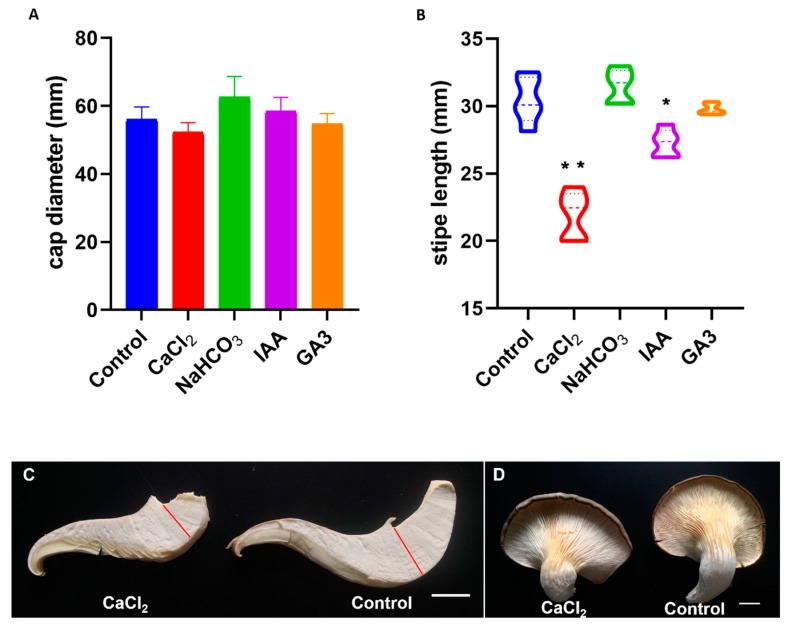
Statistical and phenotype analysis of the cap diameter and stipe length. (**A**) The cap diameter of different treatment groups. Each of the treatment showed no significant difference by Mann–Whitney test compared with the control, respectively. (**B**) The stipe length of different treatment. Both CaCl_2_ (*p*-value = 0.0079) and IAA (*p*-value = 0.0159) treatment showed a significant difference from the control by Mann–Whitney test. (**C**,**D**) Sectioning and phenotype comparison of the seven-day-grown fruiting body treated with CaCl_2_ (**C**,**D**, **left**) and control (**C**,**D**, **right**). Red lines separate the cap and stipe, respectively. Bars = 10 mm

In order to get the precise results, we set five biological replicates for each treatment, and each biological replicate included 24 bags of fruiting body grown on the medium. We first measured the diameter of the cap in each group and found that the cap diameter in each group did not show a significant difference compared with the control (Figure 7A). Then we tested the length of the stipe, and we found that the NaHCO_3_ and GA_3_ treatment group did not show significant difference (*p*-value = 0.3095 and 0.5476, respectively), while the length of stipe in both CaCl_2_ and IAA treatment group reduced greatly (*p*-value = 0.0079 and 0.0159, respectively) compared with the control (Figure 7B–D). The CaCl_2_ treatment has an even better inhibitory effect on the development of stipe than the IAA treatment. These results suggested that the exogenous application of CaCl_2_ and IAA in the early developmental stage of fruiting body can affect the primordium differentiation and stipe development, especially the effect of CaCl_2_ on inhibiting the stipe development.

**Figure 8:**
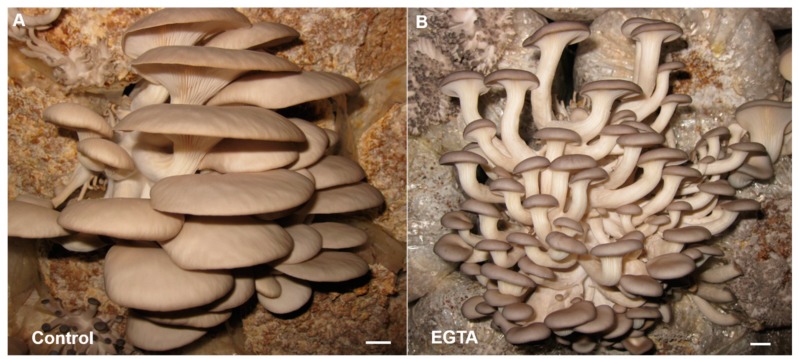
Phenotype of the primordium treated with EGTA for seven days compared with the control. Compared with the control (**A**), the EGTA-treated fruiting body (**B**) produced quite long stipe. Bars = 1 cm.

In order to figure out the mechanism of CaCl_2_ on inhibiting the stipe development, we used the calcium ion chelator EGTA to treat the primordia of the *Pleurotus ostreatus* fruiting body. EGTA is a calcium-ion-chelating agent, which can chelate the free endogenous calcium ions in cells. We found that when treated with 1 mM of EGTA, the *Pleurotus ostreatus* fruiting bodies generated quite long stipes (Figure 8A,B), which was contrary to the phenotype treated with CaCl_2_ (Figure 7C,D). This result indicated that endogenous Ca^2+^ has great influence on the primordium and its further differentiation and development, especially in the process toward stipe differentiation. The short stipe phenotype induced by CaCl_2_ treatment is probably caused by exogenous Ca^2+^ stimulation, which disrupted the original differentiation direction and made the primordium stop differentiating toward the stipe or/and more inclined to differentiate to the cap.

## 3. Discussion

The cap and stipe are different tissues, ranging from cell type to tissue structure. At the molecular level, although they share the same genome, there are great differences in gene expression, protein translation, metabolism, and even post-translational modification. Our results revealed great differences in metabolic pathways from the protein level, indicating the integrated and coordinated regulatory mechanism from the translational and metabolic level during the cap and stipe development. In the early developmental stage, signaling molecules that regulate the fruiting body differentiation may initially form these metabolites, even their derivatives, or they are transmitted to different signaling molecules, which ultimately leads to different developmental fates of cap and stipe.

### 3.1. Potential Mechanism of Ca^2+^ on Inhibiting the Stipe Growth and Differentiation

Fungi, like plants, have vacuoles and cell walls. ER and vacuole are the main calcium storage organelles in fungi [38]. The Ca^2+^ in the cytoplasm is usually kept in a stable concentration [39]. As the second messenger, Ca^2+^ participates in many processes, like growth, differentiation, and stress response. There are many kinds of external stimuli for the fungi. For example, the mechanical stimulus, hypo-osmotic stress and high external Ca^2+^ concentration, both of which can cause a short increase of cytosolic-free Ca^2+^ for hundreds of seconds, and then activate the downstream signaling pathways of fungi, to response to these external stimuli [40]. Therefore, our CaCl_2_ treatment was an external stimulus, increasing the cytosolic Ca^2+^ concentration of the primordium briefly, activating the downstream signals, and then affected the further differentiation fate of the primordium. Furthermore, we speculate that the increased cytosolic Ca^2+^ may be caused by the rapid influx of Ca^2+^ from the ER or vacuole, by affecting the permeability of Ca^2+^ channels on ER and vacuole membranes. We observed a significant increase in the membrane part in the GO enrichment (Figure 3B).

### 3.2. Sphingolipids Are Indispensable Signaling Mediators in Regulating the Cap and Stipe Differentiation

We also observed that many of the DEPs were involved in lipid metabolism, especially the sphingolipid metabolism and signaling pathways (Figure 4A,B). Membrane system, as an important structural and functional component in all kinds of cells and organelles, plays a substantial role in maintaining the specificity of the cell type or tissue. As signaling molecules, how the sphingolipids are perceived and regulated is poorly understood. Recent studies have shown that ER-localized Orm-family proteins mediate sphingolipid homeostasis [41,42]. Sphingolipids are functionally conserved but structurally diversified between species [31,43]. The enriched sphingolipid metabolism pathway between the stipe and cap indicates that there are differences in membrane structure or/and sphingolipid homeostasis between the two tissues, which is also consistent with the GO enrichment (Figure 3B). As the second messenger in eukaryotes, the Ca^2+^ pathways are evolutionally conserved in different species [44]. However, the cell structures vary between species and cell types. Therefore, a series of functionally conserved molecules with structural diversities are needed as downstream mediators of the Ca^2+^ pathways. The sphingolipids are such molecules that have conserved functions and great structural diversities between species and cell types. The localization of sphingolipid in the cells further determines its importance and necessity as signal molecules, especially in the process of cell-to-cell communication, such as the Ca^2+^ signaling transmembrane transmission. Although research about functions of the sphingolipids on Ca^2+^ signaling is limited in fungi, especially in the edible fungi, many reports have revealed that Ca^2+^ and sphingolipid signaling play a key role in many aspects of development in other model organisms [30,32,45,46,47,48,49]. In addition, there have been reports that Ca^2+^ can participate in the regulation of sphingolipid metabolism in fungi, although the molecular mechanism is obscure now [50]. The differences between stipe and cap in the sphingolipid-signaling pathway suggest that the Ca^2+^ may affect the primordium differentiation and stipe development through membrane-localized sphingolipid molecules, which can transmit the developmental signals through the membrane to downstream regulators.

## 4. Materials and Methods

### 4.1. Fungal and Culture Conditions

The *Pleurotus ostreatus* strain was from the Liaoning Center of Culture Collection (LCCC) (Liaoning, China), and the strain number is LCCC 50563. The fruiting body was cultured in the mushroom growth room, with a temperature of 20 ± 2 °C, humidity of 90~95%, carbon dioxide concentration of 550 ± 50 ppm, and light intensity of 350~500 lux. For the medium, the corn cob, bran, cornmeal, lime, and gypsum were mixed with 85:10:2:2:1 ratio (m/m), water content 60% (m/m), sterilized for 12 h, and packed into the 17 × 45 cm polyethylene bags; single bag weight was 1.25 kg.

### 4.2. Chemical Inducers Application

Inducers were diluted to their working concentration and were sprayed to the surface, at the beginning of the primordium stage, by the spray bottle, three times a day, at 08:00, 12:00, and 16:00, respectively, for 7 days, till the maturation of the fruiting body.

### 4.3. Protein Extraction, Digestion, iTRAQ Labeling, and High pH Fractionation

The extraction of the total protein, in-solution digestion and high-pH reversed-phase fractionation was followed as described previously [51]. The peptide mixture of 100 μg for each sample was labeled, using iTRAQ reagent, according to the manufacturer’s instructions (AB Sciex, Redwood City, CA, USA). The three replicates of the stipes were labeled with the isobaric tag 113, 115, and 117, and the caps were labeled with 116, 119, and 121.

### 4.4. LC–MS/MS Analysis

After fractionation, a total of 40 fractions were collected and subsequently pooled into 24 fractions according to the chromatography. Each fraction was injected for nano LC–MS/MS analysis. The peptide mixture was loaded onto a reverse-phase trap column (Thermo Scientific Acclaim PepMap100, 100 μm × 2 cm, nanoViper C18, Waltham, MA, USA) connected to the C18 reversed-phase analytical column (Thermo Scientific Easy Column, 10 cm long, 75 μm inner diameter, 3μm resin) in buffer A (0.1% formic acid) and separated with a linear gradient of buffer B (84% acetonitrile and 0.1% formic acid), at a flow rate of 300 nl/min controlled by IntelliFlow technology. The total time of each fraction was 65 min. Mass spectrometry analysis was performed on a Q Exactive mass spectrometer (Thermo Scientific) that was coupled to Easy nLC (Proxeon Biosystems, now Thermo Fisher Scientific) for 65 min. The setting of the mass spectrometer was followed as described previously [51].

### 4.5. Data Analysis

MS/MS spectra were searched by using Proteome Discoverer Software 2.1 against the species of *Pleurotus ostreatus* (13,042 sequences, downloaded 14 March 2018) from NCBI non-redundant database and the decoy database. The highest score for a given peptide mass was used to identify parent proteins. The parameters for protein searching were set as follows: tryptic digestion with up to two missed cleavages, carbamidomethylation of cysteines as the fixed modification, and oxidation of methionine and protein N-terminal acetylation as variable modifications. Peptide spectral matches were validated based on q-values, at a 1% false discovery rate (FDR). For protein identification, the peptide mass tolerance is up to 20 ppm, and the MS/MS tolerance is up to 0.1 Da. Both *p*-value < 0.05 and the ratio of stipe/cap more than 1.5 or less than 0.67 were applied to select the DEPs.

### 4.6. Bioinformatics Analysis

For the DEPs, we used the NCBI BLAST client software (ncbi-blast-2.2.28+-win32.exe) (downloaded from ftp://ftp.ncbi.nlm.nih.gov/blast/) to search the NCBI Nonredundant database, to find homologous sequences to transfer annotation to the query sequences. The top 10 blast hits of the query sequences with *E*-value less than 1e-3 were retrieved and loaded into Blast2GO (Version 2.7.2) (BioBam, Valencia, Spain) for GO2 classification. The DEPs were blasted against the Kyoto Encyclopedia of Genes and Genomes (KEGG) database in order to retrieve their KOs and mapped to the related pathways. The protein–protein interaction analysis was performed by blasting the query sequences in the STRING fungi database, with high confidence of the interaction score (0.7) in all active interaction sources.

### 4.7. RNA Extraction and Quantitative Real-Time PCR Analysis

Total RNA was extracted from the stipe and cortex of the cap of the fruiting body by the TRIeasyTM Total RNA Extraction Reagent (Yeasen, Shanghai, China), according to the manufacturer’s instructions. The concentration and quality were detected by UV spectrophotometric analysis. The reverse-transcription polymerase chain reaction was performed by First Strand cDNA Synthesis Kit ReverTra Ace -α (Toyobo, Osaka, Japan). Quantitative real-time PCR was performed on the LightCycler 96 (Roche, Basel, Switzerland), using the SYBR Green q-PCR Master Mix (Yeasen, Shanghai, China). The pep gene was used as the reference gene [52]. The primers for pep gene and the validated gene were listed in Table S6.

## 5. Conclusions

Our results revealed great differences in metabolic pathways from the protein level and the regulatory role of the Ca^2+^ in the primordium differentiation and stipe development. We also discussed the potential regulatory mechanism of Ca^2+^ on inhibiting the stipe development, as well as the indispensable role of sphingolipid as a kind of signaling molecule in mediating Ca^2+^ signaling during development. More importantly, our findings can be applied to the cultivation of the *Pleurotus ostreatus* and provide a reference for a more edible fungus.

## Figures and Tables

**Table 1:** The detailed information of proteins involved in the most significant three pathways.

Pathway Name	Accession	Annotation	Ratio Stipe/Cap	*p*-Value
Sphingolipid metabolism	646310867	KYQ37540.1 putative sphingomyelin phosphodiesterase asm-3	1.532292106	0.019700878
	646310992	KDQ32134.1 glycoside hydrolase family 27 protein	0.381533502	0.020176311
	646306316	KYQ40993.1 Sphingosine-1-phosphate lyase	0.633097441	0.000435157
	646310518	KDQ31660.1 glycoside hydrolase family 30 protein	0.568217459	0.008990541
	646310073	KYQ45887.1 Inositol phosphosphingolipids phospholipase C	0.566318538	0.002894861
Starch and sucrose metabolism	646305860	KDQ27006.1 glycoside hydrolase family 13 protein	0.602831197	0.039272074
	646308345	KDQ29489.1 glycoside hydrolase family 13 protein	0.626457034	0.040795714
	646310601	KDQ31743.1 glycosyltransferase family 20 protein	0.524911032	0.022925933
	646302526	KDQ23675.1 glycosyltransferase family 35 protein	0.540832049	0.007134693
	646301575	KDQ22726.1glycosyltransferase family 3 protein	0.659292035	0.022073266
	646308401	KDQ29545.1 glycoside hydrolase family 3 protein	1.530577815	0.006173318
	646310511	KDQ31653.1 glycoside hydrolase family 3 protein	0.586462189	0.006815785
Sphingolipid signaling pathway	646303386	AAK15758.1 ras-like protein	0.628664495	0.002764913
	646310867	KYQ37540.1 putative sphingomyelin phosphodiesterase asm-3	1.532292106	0.019700878
	646310073	KYQ45887.1 Inositol phosphosphingolipids phospholipase C	0.566318538	0.002894861
	646304707	ESK89222.1 spo14	0.651073198	0.023107308
	646301576	XP_001886453.1 heterotrimeric G-protein alpha subunit, GPA3-like protein	0.573976915	0.003752769
	646306316	KYQ40993.1 Sphingosine-1-phosphate lyase	0.633097441	0.000435157

## Supplementary Materials

Supplementary Materials can be found at https://www.mdpi.com/1422-0067/20/24/6317/s1. Table S1. Proteins that showed upregulated expression in the cap compared with the stipe. Table S2. Proteins that showed downregulated expression in the cap compared with the stipe. Table S3. Detailed information of all the identified proteins, including accession, coverage, expression level, ratio, and *p*-value. Table S4. Detailed protein information of the significantly enriched GO, including annotation, ratio, and *p*-value. Table S5. Detailed protein information of the predicted interactive partners. Table S6. Primers used in this study.
