# Supplementary material for: Comparative Proteomic Analysis of Pleurotus ostreatus Reveals Great Metabolic Differences in the Cap and Stipe Development and the Potential Role of Ca2+ in the Primordium Differentiation
Source: Int J Mol Sci. 2019 Dec 14;20(24):6317. doi: 10.3390/ijms20246317 (PMC6940972; doi:10.3390/ijms20246317)
Supplement: Supplementary file 1 [file ijms-20-06317-s001.zip › Supplementary Materials-to proofreading/Table S1.docx]

Table S1. Proteins that showed up-regulated expression in the stipe compared with the cap.

| Accession | Annotation | Coverage | Peptides | PSMs | Unique Peptides | stipe/cap | p value |
| --- | --- | --- | --- | --- | --- | --- | --- |
| 646312899 | EIW62257.1 Cerato-platanin | 17.85714 | 3 | 7 | 3 | 2.923479 | 0.039785 |
| 646308406 | XP_001880255.1 predicted protein | 13.84929 | 7 | 13 | 7 | 2.816794 | 0.015412 |
| 646303766 | XP_001877241.1 predicted protein | 22.51309 | 4 | 11 | 4 | 2.797468 | 0.0054 |
| 646310175 | KDQ31318.1 hypothetical protein PLEOSDRAFT_1075438 | 24.11765 | 4 | 7 | 4 | 2.539823 | 0.0003 |
| 646310010 | OCB85625.1 acid protease | 16.90821 | 5 | 12 | 4 | 2.529412 | 0.012488 |
| 646310418 | KDQ31561.1 carbohydrate-binding module family 13 protein | 5.806452 | 1 | 28 | 1 | 2.487507 | 0.00323 |
| 646303034 | KDQ24183.1 putative aldo-keto reductase | 38.88889 | 11 | 44 | 6 | 2.412969 | 0.002028 |
| 646308645 | KDQ29789.1 hypothetical protein PLEOSDRAFT_167611 | 3.873239 | 1 | 1 | 1 | 2.378378 | 0.025646 |
| 646310061 | XP_007848021.1 extracellular dioxygenase | 7.692308 | 2 | 8 | 2 | 2.234501 | 0.002291 |
| 646305428 | XP_001874523.1 predicted protein | 20.61856 | 6 | 14 | 6 | 2.156234 | 0.001949 |
| 646304176 | KXN90138.1 Cytochrome P450 4A4 | 19.37269 | 9 | 22 | 9 | 2.097057 | 0.0007 |
| 270056445 | CUA69353.1 Extracellular metalloprotease SMAC_06893 | 22.27273 | 3 | 15 | 3 | 2.065917 | 0.004955 |
| 646303123 | KDQ24271.1 carbohydrate esterase family 4 protein | 22.98387 | 6 | 42 | 5 | 2.015578 | 0.000244 |
| 646307944 | KZT37627.1 NAD(P)-binding protein | 45.65217 | 11 | 33 | 11 | 1.980129 | 1.23E-05 |
| 646310551 | KYQ33927.1 Cytochrome P450 4F12 | 2.466793 | 1 | 3 | 1 | 1.971768 | 0.007109 |
| 646309207 | XP_007847740.1 sterol carrier protein 2 | 24.34783 | 11 | 36 | 11 | 1.884615 | 0.000678 |
| 646310174 | KDQ31317.1 hypothetical protein PLEOSDRAFT_1088727 | 10.25641 | 1 | 1 | 1 | 1.88371 | 0.001202 |
| 646304631 | PBK74882.1 HSP20-like chaperone | 30.64516 | 6 | 22 | 6 | 1.864439 | 0.000107 |
| 646307695 | KYQ35410.1 Pyranose dehydrogenase 3 | 4.745763 | 2 | 2 | 2 | 1.834199 | 0.042838 |
| 646303656 | XP_001836391.2 fatty acid hydroxylase | 3.823529 | 1 | 2 | 1 | 1.818694 | 0.004099 |
| 646307207 | XP_001876958.1 predicted protein | 7.637655 | 4 | 7 | 4 | 1.804206 | 0.009413 |
| 646301666 | KYQ40218.1 Ubiquitin-like protein SMT3 | 14.13043 | 2 | 10 | 2 | 1.799813 | 0.008365 |
| 646311852 | KDQ32993.1 hypothetical protein PLEOSDRAFT_1051875 | 14.92537 | 6 | 9 | 6 | 1.798507 | 0.010906 |
| 646311930 | KYQ36289.1 D-tyrosyl-tRNA(Tyr) deacylase | 7.54717 | 1 | 2 | 1 | 1.794131 | 0.005609 |
| 646301320 | XP_001830890.1 small heat shock protein | 11.76471 | 2 | 10 | 1 | 1.787639 | 0.020091 |
| 646310627 | 2KJL_A Chain A, Nmr Structures Of A Designed Cyanovirin-N Homolog Lectin | 10.78431 | 1 | 19 | 1 | 1.764977 | 0.01151 |
| 646301803 | KYQ33558.1 Zinc-type alcohol dehydrogenase-like protein PB24D3.08c | 18.54839 | 6 | 21 | 6 | 1.764977 | 0.009896 |
| 646307675 | XP_001884394.1 predicted protein | 0.717213 | 1 | 1 | 1 | 1.743484 | 0.007305 |
| 646303050 | GAT50708.1 predicted protein | 8.679245 | 2 | 3 | 1 | 1.73303 | 0.009148 |
| 646307147 | GAT49086.1 predicted protein | 12.37113 | 2 | 12 | 2 | 1.724796 | 0.000653 |
| 646303781 | PAV18709.1 acetylornithine aminotransferase | 11.42241 | 4 | 4 | 4 | 1.724342 | 0.000631 |
| 646304261 | KDQ25408.1 hypothetical protein PLEOSDRAFT_1113427 | 48.82813 | 14 | 60 | 14 | 1.71448 | 0.001247 |
| 646303673 | EUC65703.1 GNAT family acetyltransferase, putative | 24.01575 | 6 | 18 | 6 | 1.703921 | 1.4E-05 |
| 646309388 | KYQ46038.1 FK506-binding protein 5 | 9.326425 | 2 | 4 | 2 | 1.701936 | 0.024186 |
| 646304353 | KYQ41335.1 Glutathione S-transferase | 21.96262 | 4 | 11 | 4 | 1.70072 | 0.008134 |
| 646311431 | XP_007856810.1 Clavaminate synthase-like protein | 23.15436 | 7 | 16 | 7 | 1.695418 | 3.46E-05 |
| 646311428 | XP_001875674.1 predicted protein | 3.614458 | 2 | 2 | 2 | 1.688172 | 0.002179 |
| 646312054 | KYQ39661.1 Trafficking protein particle complex subunit 10 | 1.73913 | 2 | 5 | 1 | 1.687416 | 0.037203 |
| 646302353 | XP_007868815.1 NAD P-binding protein | 8.28877 | 3 | 4 | 3 | 1.677822 | 0.012489 |
| 646307945 | KDQ29089.1 hypothetical protein PLEOSDRAFT_1096488 | 14.77273 | 2 | 3 | 2 | 1.674989 | 0.010851 |
| 646309084 | XP_012180418.1 predicted protein | 5.882353 | 1 | 6 | 1 | 1.666667 | 0.003895 |
| 646302301 | XP_001879490.1 predicted protein | 16.75978 | 3 | 3 | 3 | 1.659131 | 0.016103 |
| 646306384 | PAV18941.1 histone-specific chaperone chz1 | 10.28037 | 1 | 2 | 1 | 1.652078 | 0.031811 |
| 646306916 | XP_001880149.1 predicted protein | 7.710843 | 3 | 3 | 3 | 1.642731 | 0.004384 |
| 646312374 | GAT53233.1 predicted protein | 30.2521 | 3 | 4 | 3 | 1.637363 | 0.01102 |
| 646304134 | PBK59752.1 FAD-binding domain-containing protein | 6.174957 | 3 | 3 | 3 | 1.63345 | 0.017567 |
| 646305018 | KYQ34755.1 Benzoylformate decarboxylase | 1.77706 | 1 | 1 | 1 | 1.623524 | 0.009747 |
| 646301520 | XP_001889651.1 predicted protein | 29.20792 | 5 | 7 | 5 | 1.61938 | 0.02358 |
| 646306861 | KIJ19123.1 mannose-1-phosphate guanylyltransferase | 54.12262 | 21 | 86 | 21 | 1.604167 | 0.002227 |
| 646312227 | XP_001874914.1 predicted protein | 11.75497 | 7 | 12 | 7 | 1.60078 | 0.006095 |
| 646303212 | EPQ51442.1 alpha/beta-hydrolase | 26.19808 | 6 | 12 | 6 | 1.594034 | 0.001866 |
| 646310055 | KZV69303.1 Endoribonuclease L-PSP | 47.2 | 5 | 52 | 5 | 1.590674 | 0.023829 |
| 646304596 | OAX42843.1 chorismate mutase | 11.72414 | 3 | 3 | 3 | 1.589555 | 0.000455 |
| 646313051 | KZT65672.1 C4-methyl sterol oxidase | 4.833837 | 2 | 4 | 2 | 1.586207 | 0.00695 |
| 646304732 | KDQ25879.1 hypothetical protein PLEOSDRAFT_1090179 | 60.64257 | 10 | 169 | 10 | 1.583549 | 0.024761 |
| 646304130 | KZT19769.1 NAD(P)-binding protein | 41.32231 | 7 | 19 | 7 | 1.581325 | 0.039029 |
| 646302371 | KDQ23521.1 hypothetical protein PLEOSDRAFT_1090878 | 51.68067 | 10 | 154 | 10 | 1.581075 | 0.014464 |
| 646311494 | KDQ22194.1 hypothetical protein PLEOSDRAFT_1072817 | 19.06977 | 3 | 4 | 3 | 1.580645 | 0.000202 |
| 646306276 | KYQ41230.1 Lactoylglutathione lyase | 50.61728 | 8 | 39 | 8 | 1.577749 | 0.002604 |
| 646311894 | KYQ40919.1 Annexin A11 | 21.42857 | 6 | 9 | 6 | 1.560819 | 0.001891 |
| 646303432 | PCH42624.1 nicotinate phosphoribosyltransferase | 24.40191 | 10 | 21 | 10 | 1.557545 | 0.049411 |
| 646307562 | KDQ28706.1 hypothetical protein PLEOSDRAFT_156443 | 2.711864 | 1 | 5 | 1 | 1.556029 | 0.001207 |
| 646305085 | CUH74627.1 subtilisin-like peptidase | 8.776596 | 3 | 9 | 3 | 1.553427 | 0.016701 |
| 646304461 | GAT60266.1 predicted protein | 23.30097 | 4 | 7 | 4 | 1.55102 | 0.010607 |
| 646302859 | KIL63993.1 GMC oxidoreductase | 33.89545 | 21 | 89 | 20 | 1.536998 | 0.001345 |
| 646309778 | SJL08622.1 related to epoxide hydrolase | 18.78788 | 6 | 31 | 2 | 1.536998 | 0.005903 |
| 646309131 | GAW05012.1 specific transcriptional repressor | 2.036199 | 1 | 1 | 1 | 1.536152 | 0.001241 |
| 646310925 | XP_007384312.1 delta-sterol C-methyltransferase | 25.49575 | 8 | 20 | 8 | 1.533361 | 0.009304 |
| 646310867 | KYQ37540.1 putative sphingomyelin phosphodiesterase asm-3 | 8.976378 | 5 | 5 | 5 | 1.532292 | 0.019701 |
| 646308401 | KDQ29545.1 glycoside hydrolase family 3 protein | 31.64706 | 26 | 106 | 26 | 1.530578 | 0.006173 |
| 646310758 | XP_007852150.1 diamine n-acetyltransferase | 29.65116 | 6 | 21 | 6 | 1.522068 | 0.00637 |
| 646306754 | OSC97070.1 GatB/YqeY domain-containing protein | 28.96175 | 7 | 10 | 7 | 1.522068 | 0.005979 |
| 646309840 | KDQ30983.1 carbohydrate esterase family 1 protein | 36.55172 | 7 | 41 | 7 | 1.518472 | 0.004612 |
| 646310265 | XP_001873528.1 predicted protein | 38.80597 | 5 | 21 | 5 | 1.515723 | 0.019282 |
| 646303186 | GAT48717.1 predicted protein | 17.72152 | 5 | 9 | 5 | 1.514669 | 0.012605 |
| 646311773 | KYQ40799.1 putative enoyl-CoA hydratase 2 | 38.01917 | 12 | 50 | 12 | 1.51193 | 0.00667 |
| 646308097 | XP_007364376.1 NAD-aldehyde dehydrogenase | 30.01988 | 16 | 96 | 15 | 1.510879 | 0.000582 |
| 646301013 | KYQ31919.1 2-keto-3-deoxy-L-rhamnonate aldolase | 17.41294 | 3 | 8 | 3 | 1.507731 | 0.001631 |
| 646302579 | KYQ42100.1 Nucleolin 2 | 33.80282 | 9 | 27 | 9 | 1.503129 | 0.031919 |
