# Supplementary material for: Comparative Proteomic Analysis of Pleurotus ostreatus Reveals Great Metabolic Differences in the Cap and Stipe Development and the Potential Role of Ca2+ in the Primordium Differentiation
Source: Int J Mol Sci. 2019 Dec 14;20(24):6317. doi: 10.3390/ijms20246317 (PMC6940972; doi:10.3390/ijms20246317)
Supplement: Supplementary file 1 [file ijms-20-06317-s001.zip › Supplementary Materials-to proofreading/Table S2.docx]

Table S2. Proteins that showed down-regulated expression in the stipe compared with the cap.

| Accession | Annotation | Coverage | Peptides | PSMs | Unique Peptides | stipe/cap | p value |
| --- | --- | --- | --- | --- | --- | --- | --- |
| 646301940 | unknown protein | 6.486486 | 1 | 1 | 1 | 0.668985 | 0.0132 |
| 646302351 | unknown protein | 9.18197 | 4 | 10 | 4 | 0.668521 | 0.020762 |
| 646304842 | unknown protein | 36.81319 | 10 | 27 | 10 | 0.667408 | 0.000528 |
| 646303250 | GAW02139.1 dna binding protein ncp1 | 29.87288 | 16 | 64 | 16 | 0.666667 | 0.01518 |
| 646306836 | XP_001877435.1 predicted protein | 4.700855 | 1 | 2 | 1 | 0.666019 | 0.008929 |
| 646306219 | KDQ27364.1 glycoside hydrolase family 18 protein | 8.641975 | 3 | 4 | 3 | 0.665741 | 0.038712 |
| 646302170 | KYQ35918.1 Spindle pole body component alp4 | 1.77665 | 1 | 1 | 1 | 0.664355 | 0.012982 |
| 646304460 | OCH92846.1 NCA2-domain-containing protein | 10.10249 | 5 | 5 | 5 | 0.664171 | 6.86E-05 |
| 646312250 | XP_001833154.2 guanine nucleotide-binding protein subunit gamma | 36.58537 | 3 | 8 | 3 | 0.663616 | 0.000435 |
| 646305680 | KIY60985.1 histone-fold-containing protein | 50.48544 | 5 | 34 | 1 | 0.66251 | 0.004116 |
| 646301575 | KDQ22726.1 glycosyltransferase family 3 protein | 30.4878 | 17 | 59 | 17 | 0.659292 | 0.022073 |
| 158251744 | YP_001504358.1 cytochrome c oxidase subunit 1 (mitochondrion) | 1.52381 | 1 | 1 | 1 | 0.657917 | 0.00044 |
| 646312072 | KYQ43280.1 Kynurenine 3-monooxygenase | 5.964215 | 3 | 4 | 3 | 0.657277 | 0.016128 |
| 646307469 | XP_001887829.1 predicted protein | 6.944444 | 2 | 2 | 2 | 0.657001 | 0.009561 |
| 646305918 | XP_001874568.1 MFS monosaccharide transporter | 13.39623 | 5 | 10 | 5 | 0.656543 | 0.010285 |
| 646310337 | KDQ31480.1 hypothetical protein PLEOSDRAFT_1111828 | 5.603448 | 1 | 1 | 1 | 0.656362 | 0.003958 |
| 646304257 | KDQ25404.1 hypothetical protein PLEOSDRAFT_1045569 | 2.329749 | 1 | 1 | 1 | 0.656086 | 0.001178 |
| 646301540 | GAW02928.1 endosomal cargo receptor | 10.32864 | 1 | 1 | 1 | 0.655629 | 0.001742 |
| 646311664 | KDQ32805.1 hypothetical protein PLEOSDRAFT_185740 | 2.670623 | 1 | 5 | 1 | 0.654535 | 0.000188 |
| 646307004 | KYQ41036.1 ABC1 family protein MCP2 | 1.923077 | 1 | 1 | 1 | 0.65426 | 0.045755 |
| 646301587 | XP_012177738.1 predicted protein | 3.125 | 1 | 2 | 1 | 0.654079 | 0.000332 |
| 646309547 | KYQ40421.1 putative RNA-dependent RNA polymerase SHL2 | 7.841141 | 7 | 8 | 6 | 0.653444 | 0.003126 |
| 646304562 | KDQ25709.1 glycoside hydrolase family 35 protein | 5.808325 | 6 | 17 | 1 | 0.652437 | 0.038969 |
| 646303679 | KDQ24827.1 hypothetical protein PLEOSDRAFT_161137 | 9.821429 | 2 | 2 | 2 | 0.651252 | 0.014664 |
| 646301187 | KYQ34449.1 UPF0187 protein | 9.551657 | 4 | 9 | 4 | 0.651073 | 0.001486 |
| 646304707 | ESK89222.1 spo14 | 3.043801 | 4 | 4 | 4 | 0.651073 | 0.023107 |
| 646308316 | XP_001877050.1 predicted protein | 53.59116 | 9 | 48 | 8 | 0.651073 | 0.007591 |
| 646308271 | PBK72790.1 P-loop containing nucleoside triphosphate hydrolase protein | 27.42718 | 13 | 46 | 13 | 0.651073 | 0.002225 |
| 646306921 | XP_007307828.1 cytochrome P450 | 2.105263 | 1 | 2 | 1 | 0.650798 | 0.000465 |
| 646306971 | KYQ41864.1 Thioredoxin-like protein AAED1 | 37.31343 | 6 | 13 | 6 | 0.649986 | 0.000805 |
| 646309206 | KYQ41452.1 Poly [ADP-ribose] polymerase 2 | 9.033281 | 5 | 8 | 5 | 0.64989 | 0.001592 |
| 646304391 | XP_001876099.1 predicted protein | 12.25296 | 2 | 7 | 2 | 0.649711 | 0.003102 |
| 646303394 | KYQ41613.1 Trimethyllysine dioxygenase | 3.539823 | 1 | 2 | 1 | 0.649258 | 0.002563 |
| 646308092 | KYQ42625.1 Golgi apparatus membrane protein TVP38 | 3.061224 | 1 | 1 | 1 | 0.649258 | 0.000653 |
| 646301677 | KYQ40096.1 putative mitochondrial carrier C12B10.09 | 12.11268 | 5 | 8 | 5 | 0.647899 | 0.002916 |
| 646304667 | KYQ39444.1 Lactose permease | 11.63227 | 7 | 12 | 7 | 0.647899 | 0.017969 |
| 646308033 | EAU84252.1 peptidase | 32.73196 | 11 | 54 | 11 | 0.647269 | 0.000413 |
| 646305095 | KYQ41715.1 Protein rot1 | 12.5 | 2 | 4 | 2 | 0.647172 | 0.001132 |
| 646305722 | XP_001875197.1 predicted protein | 40.44586 | 11 | 85 | 11 | 0.644914 | 0.000996 |
| 646309095 | EAU93241.1 YOP1 | 19.04762 | 4 | 24 | 4 | 0.644737 | 0.000265 |
| 646310684 | XP_007850528.1 translocation protein sec62 | 7.581227 | 2 | 3 | 1 | 0.644639 | 0.012757 |
| 646309769 | KYQ41918.1 K(+)/H(+) antiporter 1 | 1.449275 | 1 | 1 | 1 | 0.643836 | 0.006208 |
| 646309810 | XP_007842642.1 carnitine acyl carnitine carrier | 19.70228 | 18 | 48 | 18 | 0.642759 | 0.000373 |
| 646310913 | KZP29306.1 putative oxalate decarboxylase/oxidase | 18.75 | 5 | 17 | 5 | 0.642486 | 0.009178 |
| 646311583 | KYQ39273.1 Hydroxymethylglutaryl-CoA lyase | 17.34104 | 5 | 11 | 5 | 0.642486 | 0.003322 |
| 646310380 | KDQ31523.1 hypothetical protein PLEOSDRAFT_1102485 | 6.115108 | 2 | 3 | 2 | 0.64231 | 0.027312 |
| 646303815 | KYQ44824.1 putative transporter C3B8.04c | 5.445545 | 5 | 7 | 5 | 0.642036 | 0.002415 |
| 646312838 | KYQ39758.1 T-cell immunomodulatory protein | 2.537313 | 2 | 2 | 2 | 0.642036 | 0.021797 |
| 646303428 | XP_001880765.1 predicted protein | 40.86022 | 7 | 10 | 7 | 0.641587 | 0.001236 |
| 646303879 | XP_007849049.1 cephalosporin esterase | 8.851224 | 6 | 20 | 5 | 0.641313 | 0.010928 |
| 646311722 | KYQ32651.1 Serine/threonine-protein kinase ppk4 | 2.270816 | 2 | 2 | 2 | 0.639519 | 0.006078 |
| 646305032 | KYQ34745.1 Carboxypeptidase S1 | 7.010309 | 3 | 4 | 3 | 0.638449 | 0.006801 |
| 646308364 | KYQ43089.1 Calcium channel YVC1 | 9.591195 | 4 | 7 | 4 | 0.638002 | 0.000104 |
| 646304789 | GAW06168.1 citrate lyase beta subunit | 12.84404 | 5 | 8 | 5 | 0.637729 | 0.002472 |
| 646312081 | OBZ75833.1 Syntaxin-like protein psy1 | 16 | 5 | 15 | 5 | 0.637108 | 0.000997 |
| 646311420 | KYQ40446.1 putative drug/proton antiporter YHK8 | 2.337662 | 1 | 1 | 1 | 0.637108 | 0.00366 |
| 646307765 | KYQ38748.1 D-xylose 1-dehydrogenase (NADP(+)) 2 | 11.83575 | 5 | 8 | 4 | 0.636041 | 0.000904 |
| 646303288 | KYQ45025.1 GTP-binding protein A | 12.35521 | 2 | 2 | 2 | 0.635596 | 0.004822 |
| 646311605 | KYQ34747.1 Protein F37C4.5 | 10.75758 | 5 | 6 | 5 | 0.635496 | 0.004152 |
| 646306380 | GAW02231.1 pali-domain-containing protein | 7.608696 | 1 | 3 | 1 | 0.635323 | 0.003433 |
| 646309636 | XP_001873202.1 predicted protein | 8.791209 | 1 | 6 | 1 | 0.634877 | 4.48E-05 |
| 646309017 | KYQ31195.1 putative peroxygenase 3 | 15.05376 | 2 | 7 | 2 | 0.634877 | 0.005288 |
| 646302033 | XP_007854006.1 calcineurin-like phosphoesterase | 9.511568 | 3 | 7 | 3 | 0.634605 | 0.024289 |
| 646305472 | KDQ26618.1 hypothetical protein PLEOSDRAFT_1089818 | 38.30645 | 11 | 48 | 11 | 0.634432 | 0.002476 |
| 646306268 | OAX40913.1 Creatinase aminopeptidase | 16.63948 | 11 | 20 | 11 | 0.633987 | 2.77E-05 |
| 646312471 | XP_012179400.1 predicted protein | 8.315098 | 4 | 6 | 4 | 0.633987 | 0.000597 |
| 646306773 | KZP34257.1 palmitoyl-protein thioesterase | 5.673759 | 1 | 2 | 1 | 0.633987 | 0.001586 |
| 646306316 | KYQ40993.1 Sphingosine-1-phosphate lyase | 14.36266 | 9 | 17 | 9 | 0.633097 | 0.000435 |
| 646305806 | OAX35453.1 DUF1640-domain-containing protein | 15.34884 | 3 | 6 | 2 | 0.631765 | 0.001089 |
| 646303746 | KDQ24894.1 polyketide synthase | 2.688787 | 4 | 4 | 4 | 0.631493 | 0.004533 |
| 646302341 | XP_007853745.1 zinc carboxypeptidase | 4.148472 | 2 | 4 | 2 | 0.631321 | 0.002638 |
| 646311830 | KDQ32971.1 heme-thiolate peroxidase | 36.2069 | 10 | 49 | 10 | 0.631049 | 4.35E-05 |
| 646308186 | KYQ42618.1 General alpha-glucoside permease | 6.09375 | 2 | 5 | 2 | 0.630606 | 0.007225 |
| 646303303 | XP_001880861.1 predicted protein | 22.64151 | 3 | 6 | 3 | 0.630435 | 0.000573 |
| 646307323 | KYQ38056.1 Mitochondrial substrate carrier family protein G | 3.424658 | 1 | 3 | 1 | 0.629821 | 2.61E-07 |
| 646303386 | AAK15758.1 ras-like protein | 52.88889 | 7 | 22 | 7 | 0.628664 | 0.002765 |
| 646312910 | KYQ40519.1 3-phytase A | 17.25352 | 9 | 28 | 9 | 0.628223 | 0.000781 |
| 646306481 | PBK74300.1 Aldo/keto reductase | 19.81132 | 7 | 17 | 4 | 0.628223 | 0.024765 |
| 646302754 | PCH43196.1 sodium/hydrogen exchanger | 1.181684 | 1 | 1 | 1 | 0.627951 | 0.005552 |
| 646308376 | XP_007767690.1 retinal short-chain dehydrogenase reductase | 8.443272 | 3 | 4 | 3 | 0.626898 | 0.000483 |
| 646308345 | KDQ29489.1 glycoside hydrolase family 13 protein | 41.91176 | 27 | 119 | 27 | 0.626457 | 0.040796 |
| 646310524 | PBK76079.1 carboxypeptidase S | 9.933775 | 6 | 7 | 6 | 0.625135 | 0.030517 |
| 646304457 | XP_001875875.1 predicted protein | 3.881279 | 1 | 1 | 1 | 0.624425 | 0.014376 |
| 646304178 | KYQ41742.1 Rho GTPase-activating protein 8 | 2.878788 | 2 | 2 | 2 | 0.624256 | 0.019788 |
| 646305350 | KDQ26496.1 hypothetical protein PLEOSDRAFT_1043519, partial | 36.09959 | 7 | 13 | 7 | 0.624087 | 0.022614 |
| 646306941 | PBK65801.1 carbohydrate esterase family 9 protein | 19.46607 | 14 | 36 | 13 | 0.623377 | 0.000349 |
| 646301436 | XP_001882733.1 predicted protein | 5.714286 | 1 | 1 | 1 | 0.623377 | 0.019197 |
| 646308349 | KYQ43108.1 UPF0676 protein | 22.28261 | 9 | 18 | 9 | 0.623106 | 0.001799 |
| 646302391 | KDQ23541.1 glycoside hydrolase family 16 protein | 13.57466 | 5 | 12 | 5 | 0.622499 | 0.003407 |
| 18857701 | CAD23442.1 putative RAD1 protein | 6.470588 | 2 | 3 | 1 | 0.622228 | 0.016309 |
| 646302527 | KDQ23676.1 hypothetical protein PLEOSDRAFT_171081 | 27.78204 | 28 | 73 | 28 | 0.62206 | 0.00172 |
| 646308157 | XP_001878422.1 predicted protein | 6.639004 | 4 | 7 | 3 | 0.621622 | 0.017123 |
| 646304441 | XP_012182835.1 predicted protein | 36.98225 | 10 | 35 | 10 | 0.621454 | 0.006197 |
| 646312152 | KDQ33293.1 hypothetical protein PLEOSDRAFT_1110502 | 10.21127 | 3 | 6 | 3 | 0.62014 | 0.001494 |
| 646308354 | OSD04664.1 homoserine acetyltransferase | 2.988506 | 1 | 2 | 1 | 0.619703 | 0.015896 |
| 646308090 | KYQ42628.1 putative trans-sulfuration enzyme | 23.03797 | 7 | 29 | 7 | 0.619433 | 0.005568 |
| 646303081 | XP_001888294.1 predicted protein | 15.44554 | 7 | 17 | 4 | 0.619433 | 0.001373 |
| 646313062 | KZP34341.1 NAD(P)-binding protein | 10.56106 | 3 | 4 | 3 | 0.619433 | 0.033797 |
| 646302390 | XP_007774031.1 YIF1-domain-containing protein | 3.636364 | 1 | 2 | 1 | 0.618226 | 0.000694 |
| 646307156 | XP_001881561.1 predicted protein | 23.56322 | 5 | 6 | 5 | 0.617853 | 0.001542 |
| 646309410 | XP_001874283.1 predicted protein | 21.37097 | 4 | 23 | 4 | 0.617687 | 0.000413 |
| 646309857 | XP_007842607.1 apolipoprotein n-acyltransferase | 17.03854 | 7 | 22 | 7 | 0.617251 | 0.000642 |
| 690563178 | AIR74634.1 putative poxc laccase transcription factor | 23.18339 | 5 | 23 | 4 | 0.617084 | 0.004913 |
| 119224834 | BAF37219.1 chitin synthase | 14.28571 | 11 | 16 | 5 | 0.616379 | 0.018471 |
| 646307805 | XP_001877126.1 predicted protein | 3.85289 | 2 | 3 | 2 | 0.615944 | 0.001616 |
| 646309053 | KDQ30196.1 hypothetical protein PLEOSDRAFT_1092036 | 23.77049 | 4 | 52 | 3 | 0.615509 | 0.045545 |
| 646310115 | EIW64204.1 Arginase/deacetylase | 10.37975 | 3 | 7 | 3 | 0.615509 | 0.002652 |
| 646306212 | KYQ43894.1 putative mitochondrial carrier C4G9.2 0c | 19.14191 | 6 | 10 | 6 | 0.612903 | 0.003847 |
| 646301235 | XP_007848045.1 nhl repeat-containing protein | 5.856833 | 2 | 2 | 2 | 0.611171 | 0.017145 |
| 646305542 | KYQ34278.1 Linoleate 10R-lipoxygenase | 37.58794 | 33 | 166 | 33 | 0.611171 | 0.026386 |
| 646306356 | KIY70653.1 organic hydroperoxide resistance protein | 22.08589 | 4 | 19 | 4 | 0.610038 | 0.003741 |
| 646303213 | OBZ69450.1 putative oxidoreductase EphD | 28.92857 | 6 | 11 | 6 | 0.608579 | 0.009718 |
| 646304551 | KYQ41958.1 putative serine protease EDA2 | 24.68007 | 9 | 23 | 9 | 0.608148 | 0.003331 |
| 646312003 | OAL52280.1 acyl-CoA dehydrogenase NM domain-like protein | 11.13861 | 5 | 5 | 5 | 0.608148 | 0.0034 |
| 646301200 | GAT55761.1 predicted protein | 2.777778 | 1 | 1 | 1 | 0.608148 | 0.021343 |
| 646309568 | OJT03022.1 Altered inheritance of mitochondria protein 6 -like protein | 3.883495 | 1 | 1 | 1 | 0.607717 | 0.00782 |
| 646310224 | XP_001873481.1 predicted protein | 12.95181 | 5 | 10 | 5 | 0.607554 | 0.000773 |
| 646309327 | KDQ30470.1 carbohydrate esterase family 4 protein | 1.927195 | 1 | 1 | 1 | 0.606856 | 0.048028 |
| 646305459 | KYQ38940.1 Centromere/kinetochore protein zw10 | 3.662258 | 4 | 4 | 4 | 0.606856 | 0.019981 |
| 646311637 | XP_001879021.1 predicted protein | 7.798165 | 1 | 1 | 1 | 0.605834 | 0.042501 |
| 646307835 | XP_001876668.1 predicted protein | 4.522613 | 1 | 4 | 1 | 0.605566 | 0.009703 |
| 646306701 | KDQ27846.1 hypothetical protein PLEOSDRAFT_1083804 | 13.36032 | 4 | 9 | 4 | 0.605136 | 0.002856 |
| 646303472 | XP_009545544.1 inorganic phosphate transporter | 21.57221 | 10 | 35 | 10 | 0.604975 | 0.042423 |
| 646308718 | XP_001874376.1 predicted protein | 22.75641 | 4 | 8 | 4 | 0.603849 | 0.002156 |
| 646305860 | KDQ27006.1 glycoside hydrolase family 13 protein | 18.79321 | 30 | 74 | 30 | 0.602831 | 0.039272 |
| 646301190 | KDQ22341.1 hypothetical protein PLEOSDRAFT_177271 | 8.730159 | 1 | 2 | 1 | 0.602564 | 0.025629 |
| 646303293 | KYQ45025.1 GTP-binding protein A | 3.529412 | 1 | 1 | 1 | 0.602403 | 0.000287 |
| 646301900 | KLO17852.1 vesicle transport v-snare protein vti1 | 16.59751 | 3 | 8 | 3 | 0.600854 | 1.21E-05 |
| 646301722 | KDQ22873.1 DyP-type peroxidase | 9.325397 | 3 | 4 | 3 | 0.599573 | 0.010788 |
| 646311423 | KYQ40634.1 Zinc transporter 6-B | 2.096178 | 1 | 1 | 1 | 0.598029 | 0.001498 |
| 646302912 | GAW09154.1 Dimeric alpha+beta barrel | 45.16129 | 5 | 8 | 5 | 0.597444 | 0.009233 |
| 646304813 | XP_012183660.1 predicted protein | 14.72868 | 2 | 10 | 2 | 0.595745 | 0.023918 |
| 646305272 | KXN86207.1 Mitochondrial carnitine/acylcarnitine carrier protein CACL | 3.97351 | 1 | 1 | 1 | 0.595745 | 0.002332 |
| 646306937 | PBK74992.1 carbohydrate esterase family 9 protein | 12.51462 | 11 | 32 | 10 | 0.594049 | 0.000739 |
| 646308030 | KNZ73872.1 Dolichyl-P-Man:Man(5)GlcNAc(2)-PP-dolichyl mannosyltransferase | 29.49153 | 7 | 11 | 7 | 0.594049 | 0.000834 |
| 646307939 | KYQ33704.1 Homoisocitrate dehydrogenase | 37.60446 | 12 | 24 | 12 | 0.593625 | 0.000829 |
| 646307861 | KYQ42514.1 Protein BMH2 | 13.56784 | 3 | 29 | 2 | 0.591934 | 0.027125 |
| 646303044 | OJA08090.1 57 | 20.60302 | 3 | 15 | 3 | 0.591669 | 0.000414 |
| 646310507 | KLO10910.1 FAD-binding domain-containing protein | 1.785714 | 1 | 1 | 1 | 0.590668 | 0.000334 |
| 646310212 | GAW09688.1 cytochrome-b5 reductase | 27.13415 | 10 | 32 | 10 | 0.589669 | 2.34E-05 |
| 646306369 | GAT44505.1 predicted protein | 9.558824 | 2 | 3 | 2 | 0.588983 | 0.0091 |
| 646307637 | KYQ36526.1 putative epoxide hydrolase | 19.95614 | 7 | 19 | 7 | 0.588562 | 0.000114 |
| 646310511 | KDQ31653.1 glycoside hydrolase family 3 protein | 13.24786 | 8 | 23 | 8 | 0.586462 | 0.006816 |
| 646310617 | KIY64554.1 auxin efflux carrier | 1.492537 | 1 | 1 | 1 | 0.586462 | 0.019764 |
| 646303969 | XP_001888159.1 predicted protein | 21.56863 | 2 | 4 | 2 | 0.585778 | 0.008085 |
| 646302548 | KNZ78110.1 Protein dml-1 | 39.21569 | 7 | 11 | 7 | 0.585469 | 0.007003 |
| 646306264 | PBK79475.1 NAD(P)-binding protein | 5.985915 | 2 | 3 | 2 | 0.585359 | 0.023423 |
| 646302644 | PBK71589.1 pali-domain-containing protein | 26.75439 | 4 | 22 | 4 | 0.584104 | 0.008751 |
| 646305194 | KYQ35391.1 putative aminotransferase | 5.594406 | 3 | 6 | 3 | 0.583267 | 0.018341 |
| 646307973 | KYQ33617.1 Aspartate--tRNA ligase, mitochondrial | 12.79762 | 8 | 13 | 8 | 0.583113 | 1.33E-05 |
| 646312278 | GAW01366.1 acetamidase regulatory protein | 0.835946 | 1 | 1 | 1 | 0.581028 | 0.001875 |
| 646300927 | BAP27863.1 serine aminopeptidase | 4.017857 | 3 | 13 | 1 | 0.579363 | 0.043864 |
| 646305437 | XP_001875140.1 phosphatidic acid phosphatase | 3.809524 | 1 | 1 | 1 | 0.579363 | 0.005827 |
| 646305651 | XP_007845781.1 gly-x carboxypeptidase | 24.10256 | 11 | 26 | 8 | 0.578947 | 0.000351 |
| 646307789 | KYQ32881.1 putative membrane protein | 6.47482 | 2 | 4 | 2 | 0.578684 | 0.007668 |
| 646309247 | KDQ30390.1 glycoside hydrolase family 72 protein | 15.12456 | 6 | 27 | 6 | 0.578117 | 0.006501 |
| 646302853 | OAX32675.1 B-cell receptor-associated 31-like protein | 17.56098 | 4 | 20 | 4 | 0.576044 | 0.002363 |
| 646301576 | XP_001886453.1 heterotrimeric G-protein alpha subunit, GPA3-like protein | 3.932584 | 1 | 1 | 1 | 0.573977 | 0.003753 |
| 646303614 | XP_006458837.1 ctg1 protein | 39.63415 | 5 | 12 | 5 | 0.570681 | 0.038418 |
| 646305560 | PBK72406.1 alpha beta-hydrolase | 6.933744 | 3 | 5 | 3 | 0.570681 | 0.015627 |
| 646309322 | EPQ61056.1 tricalbin | 35.69024 | 46 | 154 | 46 | 0.569859 | 0.002638 |
| 646311935 | SJL12289.1 related to Acylpyruvase FAHD1, mitochondrial | 27.38589 | 6 | 21 | 6 | 0.569038 | 6.38E-05 |
| 646304115 | OAX35467.1 PLP-dependent transferase | 6.134969 | 1 | 1 | 1 | 0.569038 | 0.001852 |
| 646308306 | XP_007846246.1 scf e3 ubiquitin ligase complex f-box protein grra | 1.108871 | 1 | 1 | 1 | 0.568776 | 0.006069 |
| 646307287 | OBZ70072.1 Fumarylacetoacetate hydrolase domain-containing protein 2 | 34.65347 | 8 | 19 | 8 | 0.568479 | 0.000336 |
| 646310518 | KDQ31660.1 glycoside hydrolase family 30 protein | 2.772277 | 1 | 2 | 1 | 0.568217 | 0.008991 |
| 646309262 | KIY67604.1 NAD-P-binding protein | 63.34842 | 12 | 26 | 12 | 0.567398 | 0.004459 |
| 646309523 | XP_001873859.1 predicted protein | 4.918033 | 2 | 9 | 2 | 0.566989 | 0.002481 |
| 646310073 | KYQ45887.1 Inositol phosphosphingolipids phospholipase C | 9.029345 | 4 | 12 | 4 | 0.566319 | 0.002895 |
| 646306852 | XP_007847003.1 rho small monomeric gtpase | 21.33333 | 4 | 17 | 4 | 0.566023 | 0.003221 |
| 646310480 | Q6Y5M5.1 RecName: Full=Mitochondrial intermediate peptidase; Short=MIP; AltName: Full=Octapeptidyl aminopeptidase; Flags: Precursor | 1.528662 | 1 | 1 | 1 | 0.565762 | 0.024926 |
| 646312592 | XP_001878302.1 predicted protein | 2.028986 | 1 | 1 | 1 | 0.565501 | 0.002007 |
| 646307307 | XP_007365218.1 general substrate transporter | 6.967985 | 3 | 3 | 3 | 0.565354 | 0.012145 |
| 646302317 | PBK64813.1 mitochondrial carrier | 35.7377 | 11 | 39 | 11 | 0.565093 | 0.000819 |
| 646301122 | XP_007848398.1 copper radical oxidase | 10.52632 | 6 | 24 | 6 | 0.56276 | 0.043458 |
| 646301777 | XP_007330585.1 histidine phosphotranseferase | 22.47191 | 4 | 9 | 3 | 0.56276 | 0.000424 |
| 646311975 | KDQ33116.1 glycoside hydrolase family 5 protein | 9.236948 | 6 | 14 | 6 | 0.5625 | 0.005816 |
| 646312850 | KYQ39718.1 Elongation factor 3 | 33.3018 | 36 | 142 | 36 | 0.561687 | 0.026645 |
| 646301012 | XP_001882714.1 hydrophobic surface binding protein | 8.55615 | 1 | 1 | 1 | 0.560614 | 0.000109 |
| 646310391 | XP_001829415.1 CMGC/CDK protein kinase | 4.166667 | 2 | 3 | 2 | 0.558442 | 0.002323 |
| 646302885 | SJL17633.1 related to Sterol-4-alpha-carboxylate 3-dehydrogenase, decarboxylating | 27.42857 | 8 | 18 | 8 | 0.557488 | 3.59E-05 |
| 646302249 | XP_007845960.1 vesicle-associated membrane protein | 29.01786 | 7 | 23 | 7 | 0.556969 | 0.013838 |
| 646310026 | XP_007763599.1 NIPSNAP-domain-containing protein | 64.61538 | 7 | 21 | 7 | 0.556017 | 0.000163 |
| 646301165 | OCH93325.1 amidase signature enzyme | 12.95337 | 6 | 9 | 6 | 0.555757 | 8.92E-05 |
| 646307265 | PBK76829.1 putative zinc metalloprotease | 6.683168 | 5 | 9 | 5 | 0.554404 | 0.002184 |
| 646302739 | KDQ23888.1 hypothetical protein PLEOSDRAFT_1048324, partial | 6.571429 | 2 | 2 | 2 | 0.553743 | 0.014266 |
| 646305003 | KNZ75499.1 Dimethylaniline monooxygenase [N-oxide-forming] 2 | 5.141844 | 3 | 4 | 3 | 0.552393 | 0.000785 |
| 646303433 | KDQ24581.1 glycoside hydrolase family 79 protein | 12.6195 | 7 | 36 | 7 | 0.551992 | 0.016434 |
| 646306247 | KYQ33152.1 Lysosomal aspartic protease | 10.15762 | 4 | 22 | 4 | 0.551448 | 0.000186 |
| 646309259 | XP_007854469.1 dicarboxylic acid transporter | 4.152249 | 1 | 1 | 1 | 0.550788 | 0.008477 |
| 646307953 | XP_001874727.1 acetylornithine aminotransferase | 22.34763 | 9 | 39 | 9 | 0.550788 | 0.000215 |
| 646303274 | XP_007367894.1 cytochrome P450 | 21.17647 | 9 | 17 | 9 | 0.541624 | 0.006988 |
| 646305135 | KYQ35484.1 Glycolipid transfer protein B | 12.13592 | 3 | 10 | 3 | 0.541228 | 0.002043 |
| 646302526 | KDQ23675.1 glycosyltransferase family 35 protein | 36.53179 | 26 | 148 | 26 | 0.540832 | 0.007135 |
| 646310020 | XP_001874029.1 predicted protein | 28.125 | 19 | 70 | 19 | 0.540436 | 0.001141 |
| 646306454 | KYQ41143.1 Gamma-glutamyltranspeptidase 1 | 15.63025 | 8 | 18 | 8 | 0.539902 | 0.004125 |
| 646305424 | XP_001874481.1 predicted protein | 4.863222 | 1 | 1 | 1 | 0.539113 | 0.003171 |
| 646306499 | XP_007847041.1 carboxypeptidase cpds | 11.46026 | 5 | 20 | 5 | 0.538462 | 0.008581 |
| 646308607 | KDQ29751.1 PHO4 superfamily | 1.763668 | 1 | 3 | 1 | 0.537279 | 0.031371 |
| 646312369 | KYQ43237.1 NAD(P) transhydrogenase, mitochondrial | 21.32425 | 22 | 65 | 22 | 0.536885 | 0.000107 |
| 646312448 | KYQ39821.1 Zinc-type alcohol dehydrogenase-like protein C16A3.02c | 9.116809 | 3 | 4 | 3 | 0.536885 | 0.005526 |
| 646310864 | XP_001881010.1 multidrug resistance-associated ABC transporter | 3.150599 | 4 | 6 | 4 | 0.535056 | 0.004448 |
| 646306709 | XP_012179967.1 predicted protein | 22.52011 | 6 | 15 | 6 | 0.534664 | 0.001481 |
| 646303252 | KYQ45025.1 GTP-binding protein A | 23.77049 | 7 | 11 | 7 | 0.534134 | 0.007195 |
| 646305501 | KXN92417.1 Leucine aminopeptidase 1 | 26.13333 | 6 | 39 | 6 | 0.532312 | 0.031628 |
| 646303336 | BAB43911.1 probable metabolite transporter | 2.439024 | 1 | 1 | 1 | 0.531649 | 0.001142 |
| 646306793 | KYQ43949.1 Heavy metal tolerance protein | 25.33784 | 14 | 44 | 13 | 0.529442 | 0.002029 |
| 646308368 | KYQ42384.1 Glutamate carboxypeptidase 2 | 32.8844 | 21 | 51 | 21 | 0.527361 | 9.96E-06 |
| 646301522 | XP_001883372.1 predicted protein | 15.14914 | 15 | 20 | 15 | 0.526718 | 0.000185 |
| 646301766 | KDQ22917.1 carbohydrate-binding module family 13 protein | 14.87603 | 3 | 9 | 3 | 0.526584 | 0.011001 |
| 646306412 | KDQ27557.1 glycoside hydrolase family 92 protein | 12.78481 | 8 | 23 | 8 | 0.525553 | 0.000652 |
| 646310601 | KDQ31743.1 glycosyltransferase family 20 protein | 27.06767 | 20 | 74 | 20 | 0.524911 | 0.022926 |
| 646309160 | GAT48164.1 predicted protein | 18.98148 | 3 | 9 | 3 | 0.524778 | 0.011458 |
| 646306310 | KDQ27455.1 cytochrome c peroxidase | 31.38298 | 9 | 29 | 9 | 0.524644 | 0.001636 |
| 646305491 | OBZ74242.1 putative J domain-containing protein C4H3.01 | 6.698565 | 2 | 3 | 2 | 0.524524 | 0.023341 |
| 646301016 | KYQ31923.1 Cystathionine gamma-lyase | 12.22222 | 5 | 10 | 4 | 0.522843 | 0.006575 |
| 646311188 | GAT48502.1 predicted protein | 0.895857 | 1 | 6 | 1 | 0.521552 | 0.006971 |
| 646302247 | XP_007870172.1 glycerol-3-phosphate 1-acyltransferase | 3.207547 | 1 | 1 | 1 | 0.519625 | 0.01158 |
| 646312296 | XP_002469581.1 predicted protein | 33.19149 | 6 | 14 | 6 | 0.51835 | 0.003998 |
| 646301668 | GAT52610.1 predicted protein | 10.37736 | 1 | 3 | 1 | 0.51835 | 0.000179 |
| 646303080 | KYQ31782.1 Psi-producing oxygenase A | 46.55493 | 42 | 280 | 37 | 0.517704 | 0.000385 |
| 646306449 | KDQ27594.1 glycoside hydrolase family 76 protein | 5.337079 | 1 | 2 | 1 | 0.516684 | 0.005589 |
| 646308455 | KYQ43083.1 K(+)/H(+) antiporter 1 | 5.922551 | 5 | 6 | 5 | 0.515917 | 0.020731 |
| 646308323 | XP_001876593.1 GPI-anchored small secreted protein | 11.70213 | 1 | 6 | 1 | 0.514005 | 0.004086 |
| 646302009 | XP_001877852.1 predicted protein | 1.001669 | 1 | 1 | 1 | 0.513875 | 0.010442 |
| 646304209 | XP_001885770.1 predicted protein | 33.83459 | 8 | 33 | 7 | 0.510574 | 0.026975 |
| 646303010 | KDQ24159.1 hypothetical protein PLEOSDRAFT_1090690 | 51.27273 | 43 | 167 | 42 | 0.507665 | 0.030276 |
| 646301937 | OAX37417.1 bifunctional acetylglutamate kinase/N-acetyl-gamma-glutamyl-phosphate reductase | 36.99095 | 28 | 163 | 28 | 0.507665 | 2.8E-05 |
| 646301776 | OAL49031.1 P-loop containing nucleoside triphosphate hydrolase protein | 30.91787 | 6 | 20 | 6 | 0.506402 | 0.002515 |
| 646305774 | XP_001878706.1 predicted protein | 5.669291 | 4 | 4 | 4 | 0.505646 | 0.007538 |
| 315190039 | BAD16584.1 lectin | 23.0563 | 5 | 32 | 3 | 0.504891 | 0.002647 |
| 646307240 | XP_007849224.1 vacuolar protein | 19.6748 | 11 | 24 | 10 | 0.504136 | 0.038907 |
| 646308247 | XP_001876715.1 predicted protein | 8.673469 | 3 | 4 | 3 | 0.501627 | 0.002353 |
| 646311817 | KDQ32958.1 hypothetical protein PLEOSDRAFT_1098941 | 4.830918 | 2 | 7 | 2 | 0.501502 | 0.0006 |
| 646310857 | KDQ31999.1 hypothetical protein PLEOSDRAFT_1111045 | 13.11953 | 9 | 34 | 9 | 0.5 | 0.002959 |
| 646308728 | XP_001874540.1 predicted protein | 15.78947 | 3 | 3 | 3 | 0.496509 | 0.002854 |
| 646309951 | KDQ31094.1 carbohydrate esterase family 12 protein | 7.874016 | 2 | 4 | 2 | 0.496259 | 0.038559 |
| 646307337 | KYQ38137.1 Testicular acid phosphatase | 42.06897 | 13 | 461 | 13 | 0.496136 | 0.000436 |
| 646301062 | XP_007848398.1 copper radical oxidase | 11.11111 | 6 | 9 | 5 | 0.494396 | 0.000379 |
| 646301943 | KDQ23093.1 hypothetical protein PLEOSDRAFT_1090725, partial | 26.61871 | 3 | 3 | 3 | 0.491054 | 0.007768 |
| 646305164 | KDQ26310.1 hypothetical protein PLEOSDRAFT_1056930 | 8.695652 | 1 | 7 | 1 | 0.488343 | 0.000116 |
| 646305176 | CAA06292.1 laccase | 26.64165 | 11 | 32 | 2 | 0.479655 | 0.009941 |
| 646309854 | XP_001873358.1 predicted protein | 23.18339 | 20 | 51 | 20 | 0.478197 | 0.009127 |
| 646303649 | KDQ24797.1 carbohydrate-binding module family 13 protein | 11.2462 | 3 | 12 | 3 | 0.477833 | 0.000327 |
| 646305690 | KTB31094.1 putative subtilisin-like protein | 10.71429 | 5 | 29 | 5 | 0.476976 | 0.01002 |
| 646308565 | KYQ43038.1 Tripeptidyl-peptidase sed2 | 11.72414 | 6 | 35 | 6 | 0.475535 | 0.011121 |
| 646303873 | GAV99118.1 ph domain-containing protein | 21.36752 | 5 | 13 | 5 | 0.475289 | 0.004905 |
| 646304796 | KYQ34766.1 4-coumarate--CoA ligase-like 7 | 21.98582 | 8 | 20 | 8 | 0.472031 | 0.000747 |
| 646308161 | XP_002475745.1 candidate carbohydrate esterase protein from family CE9 | 21.44444 | 16 | 65 | 16 | 0.472031 | 0.000694 |
| 646312724 | SJL04742.1 related to Endothelin-converting enzyme 1 | 22.96296 | 16 | 64 | 16 | 0.470228 | 0.000708 |
| 646310797 | KDQ31939.1 glycoside hydrolase family 47 protein | 29.62963 | 11 | 90 | 9 | 0.468543 | 0.00144 |
| 646307286 | XP_001889707.1 predicted protein | 12.82051 | 11 | 53 | 4 | 0.467466 | 5.99E-05 |
| 646309620 | KDQ30763.1 polysaccharide lyase family 8 protein | 22.36287 | 15 | 50 | 14 | 0.46152 | 0.000121 |
| 646301029 | PBK94805.1 kinase-like protein | 6.374502 | 1 | 1 | 1 | 0.458901 | 0.008378 |
| 646306516 | XP_007361818.1 UTP-glucose-1-phosphate uridylyltransferase | 2.272727 | 1 | 1 | 1 | 0.458901 | 0.000366 |
| 646307143 | KDQ28288.1 glycoside hydrolase family 28 protein | 2.820513 | 1 | 1 | 1 | 0.458678 | 0.026779 |
| 646311593 | KTB29568.1 putative mannose-6-phosphatase | 23.34385 | 5 | 17 | 5 | 0.453951 | 0.00021 |
| 646303259 | KDQ24407.1 glycoside hydrolase family 10 protein | 12.99094 | 4 | 17 | 4 | 0.447768 | 0.000392 |
| 646305156 | KDQ26302.1 hypothetical protein PLEOSDRAFT_1089724 | 13.04348 | 1 | 9 | 1 | 0.444391 | 0.043774 |
| 646306824 | KYQ37511.1 Protein AIM2 | 3.333333 | 1 | 3 | 1 | 0.443696 | 1.49E-05 |
| 646312761 | XP_007301276.1 FAD/NAD-P-binding domain-containing protein | 7.966102 | 4 | 9 | 4 | 0.439434 | 0.001796 |
| 646309969 | XP_001831685.1 histone H4 | 50.48544 | 5 | 31 | 1 | 0.438159 | 0.00413 |
| 646307291 | XP_001889707.1 predicted protein | 10.99612 | 9 | 40 | 2 | 0.435063 | 0.001404 |
| 646312792 | KDQ33933.1 glycoside hydrolase family 88 protein | 25.06329 | 8 | 77 | 8 | 0.43472 | 0.00208 |
| 646304486 | KYQ45025.1 GTP-binding protein A | 4.411765 | 1 | 1 | 1 | 0.427212 | 0.025651 |
| 646304354 | XP_012185020.1 predicted protein | 1.298701 | 1 | 1 | 1 | 0.413095 | 0.004943 |
| 646301035 | KIM82116.1 Glucooligosaccharide oxidase | 6.877323 | 3 | 3 | 3 | 0.411101 | 0.005904 |
| 646305634 | XP_007845781.1 gly-x carboxypeptidase | 23.63946 | 10 | 30 | 7 | 0.40426 | 7.94E-05 |
| 646307062 | XP_009553320.1 fasciclin-like protein | 5.940594 | 1 | 7 | 1 | 0.399907 | 0.000435 |
| 646302264 | KYQ38430.1 NADPH-dependent conjugated polyketone reductase C1 | 24.25249 | 7 | 14 | 6 | 0.399347 | 0.000537 |
| 646302661 | OCH88511.1 sure-like protein | 8.852459 | 2 | 7 | 2 | 0.399021 | 0.008746 |
| 646303635 | PBK88038.1 cytochrome P450 | 3.688525 | 1 | 4 | 1 | 0.396973 | 0.030053 |
| 646308326 | XP_001876596.1 predicted protein | 1.808786 | 1 | 1 | 1 | 0.395765 | 2.43E-05 |
| 646310859 | KZV65998.1 GroES-like protein | 26.96335 | 8 | 29 | 8 | 0.386873 | 0.015676 |
| 646306864 | XP_007849647.1 late embryogenesis abundant protein | 30.41667 | 8 | 40 | 7 | 0.386642 | 0.000411 |
| 646306990 | KDQ28135.1 carbohydrate esterase family 1 protein | 22.08589 | 4 | 8 | 4 | 0.383445 | 0.000845 |
| 646309884 | KYQ46146.1 PI-PLC X domain-containing protein | 4.359673 | 1 | 1 | 1 | 0.383445 | 0.016063 |
| 646310992 | KDQ32134.1 glycoside hydrolase family 27 protein | 3.084833 | 1 | 2 | 1 | 0.381534 | 0.020176 |
| 646304087 | KDQ25234.1 polysaccharide lyase family 1 protein | 9.463722 | 2 | 4 | 2 | 0.375602 | 0.001756 |
| 646308635 | KDQ29779.1 copper-amine-oxidase superfamily | 10.94296 | 8 | 15 | 8 | 0.369238 | 0.002616 |
| 646310929 | KYQ31782.1 Psi-producing oxygenase A | 33.61169 | 14 | 57 | 11 | 0.363946 | 0.011441 |
| 646311469 | OSD00512.1 chondroitin AC/alginate lyase | 11.66667 | 6 | 12 | 6 | 0.351351 | 0.000681 |
| 646301915 | KZP26769.1 NAD(P)-binding protein | 11.57025 | 2 | 2 | 2 | 0.348618 | 0.019487 |
| 646310928 | KYQ31782.1 Psi-producing oxygenase A | 40.23669 | 4 | 39 | 2 | 0.335708 | 0.012065 |
| 646311450 | XP_007852106.1 cysteine-rich secreted protein | 5.347594 | 2 | 19 | 2 | 0.331262 | 0.02834 |
| 646303180 | KNZ73921.1 O-methylsterigmatocystin oxidoreductase | 2.33463 | 1 | 3 | 1 | 0.32714 | 0.019638 |
| 646307824 | BAD16584.1 lectin | 13.89646 | 3 | 52 | 1 | 0.31183 | 0.002027 |
| 646303209 | ELU39118.1 Hemopexin domain-containing protein | 54.43038 | 12 | 414 | 12 | 0.299329 | 2.36E-05 |
| 646305265 | EDR14453.1 agmatinase | 18.34171 | 5 | 11 | 5 | 0.298356 | 0.000535 |
| 646307640 | XP_007848398.1 copper radical oxidase | 14.7541 | 5 | 21 | 4 | 0.296801 | 0.001273 |
| 646304424 | KYQ41931.1 Alkali-sensitive linkage protein 1 | 12.54902 | 3 | 10 | 3 | 0.277411 | 0.011117 |
| 646308687 | KDQ29831.1 glycoside hydrolase family 16 protein | 3.836317 | 1 | 1 | 1 | 0.268019 | 0.003493 |
| 646305022 | KDQ26169.1 hypothetical protein PLEOSDRAFT_1090236 | 52.30769 | 3 | 27 | 3 | 0.240951 | 0.014003 |
| 646309220 | XP_001873306.1 predicted protein | 1.336303 | 1 | 1 | 1 | 0.221001 | 0.001644 |
